# Return Migrants’ Experience of Access to Care in Corrupt Healthcare Systems: The Bosnian Example

**DOI:** 10.3390/ijerph13090924

**Published:** 2016-09-19

**Authors:** Line Neerup Handlos, Karen Fog Olwig, Ib Christian Bygbjerg, Marie Norredam

**Affiliations:** 1 Danish Research Centre for Migration, Ethnicity and Health, Section for Health Services Research, Department of Public Health, University of Copenhagen, Copenhagen 1014, Denmark; mano@sund.ku.dk; 2 Department of Anthropology, University of Copenhagen, Copenhagen 1353, Denmark; karen.fog.olwig@anthro.ku.dk; 3 Section of Global Health, Department of Public Health, University of Copenhagen, Copenhagen 1014, Denmark; iby@sund.ku.dk; 4 Section of Immigrant Medicine, Department of Infectious Diseases, University Hospital Hvidovre, Hvidovre 2650, Denmark

**Keywords:** corruption, return migration, access to healthcare, Bosnia and Herzegovina

## Abstract

Equal and universal access to healthcare services is a core priority for a just health system. A key societal determinant seen to create inequality in access to healthcare is corruption in the healthcare system. How return migrants’ access to healthcare is affected by corruption is largely unstudied, even though return migrants may be particularly vulnerable to problems related to corruption due to their period of absence from their country of origin. This article investigates how corruption in the healthcare sector affects access to healthcare for refugees who repatriated to Bosnia, a country with a high level of corruption, from Denmark, a country with a low level of corruption. The study is based on 18 semi-structured interviews with 33 refugees who returned after long-term residence in Denmark. We found that the returned refugees faced greater problems with corruption than was the case for those who had not left the country, as doctors considered them to be better endowed financially and therefore demanded larger bribes from them than they did from those who had remained in Bosnia. Moreover, during their stay abroad the returnees had lost the connections that could have helped them sidestep the corruption. Returned refugees are thus particularly vulnerable to the effects of corruption.

## 1. Introduction

Equal and universal access to healthcare services is a core priority in an egalitarian health system. Factors traditionally described as barriers to access to healthcare are typically individual determinants such as age, gender, socio-economic status, geographical location, and minority affiliation. Likewise, societal determinants such as health policies, the resources available for healthcare, and the organization of the healthcare system have been described as important factors influencing access [1,2,3]. A key societal determinant which has been seen to create inequity in access to healthcare is corruption in the healthcare system [4,5,6,7]. High levels of corruption have consistently been shown to have a negative impact on population health and social well-being [8,9,10]. The negative impact of corruption on population health has been explained in terms of various societal problems resulting from corruption. Examples of causal pathways detrimental to population health are lack of economic growth, lack of properly functioning water and sanitation systems, absenteeism among healthcare personnel as well as the diversion of money from social services into private pockets and citizens’ unwillingness to pay taxes, both of which result in underfunding of the public health system [8,11]. Furthermore, high levels of corruption are related to economic inequality and lack of social capital, social cohesion and social trust, factors which in numerous contexts have been shown to be linked to poor health in the affected populations [12,13,14,15].

According to a widely accepted definition, “corruption is the misuse or abuse of public power for private gain” [16] (p. 6). Following this definition, a doctor in a public position who in any way accepts gifts or asks for extra payment as a prerequisite for giving treatment is considered to be involved in corrupt practices.

Corruption in the healthcare sector has been explored in numerous settings [7,17,18,19,20,21], with a special focus on the post-communist countries in Eastern and Central Europe and the former Soviet Union States [4,5,6,22,23,24]. One of these countries is Bosnia and Herzegovina (hereafter referred to as Bosnia) which is known to be one of the most corrupt countries in Europe [21]. High levels of corruption are common in many post-communist countries that are in economic and political transition, and in the former Yugoslavia the war and its aftermath further complicate the situation [25]. In 2011 the United Nations’ Office on Drugs and Crime found that corruption was considered to be the fourth most important problem in Bosnia: 20% of the adult population had been either directly or indirectly exposed to demands for bribery during the past twelve months. More than half of those citizens with recent experience of corruption had given bribes to doctors, and more than 60% of the adult population said that corruption occurs frequently in public hospitals [26]. Transparency International supports the portrayal of Bosnia as a state with widespread corruption by reporting that 77% of a representative sample thought that politicians are corrupt or very corrupt [27]. In contrast to Bosnia, Denmark is considered to be one of the least corrupt countries in the world. Cases of nepotism and embezzlement have occurred recently, but they are rare, and along with the other Nordic countries, Denmark ranks at the top of the global list when it comes to the absence of corruption [21].

Despite the high level of corruption in Bosnia, many Bosnian refugees have returned voluntarily to the country. During the Balkan war in the 1990s, more than two million people fled their homes, a million of whom left the country [28]. Around 17,000 fled to Denmark, where they were all eventually granted asylum [29]. By 2015, however, about 2700 individuals had returned to Bosnia [30]. In total around one million refugees had returned to their pre-war place of residence by the end of 2014, though this repatriation was not always voluntary [28]. The vast majority moved back as part of a Danish repatriation program offering 19,000 U.S. dollars (USD) as a one-off payment, medical support for the first year and monthly payments of either 520 USD for five years or 420 USD for life for everyone above 55 years of age [31]. In comparison, the average monthly salary in Bosnia is 390 USD (gross national income per capita as reported by the World Bank [32]).

A previous study has shown that elderly, chronically ill Bosnian returnees experience general well-being and high levels of satisfaction upon their return. However, they also express concerns relating to their access to health care. The returnees do not have access to healthcare services as regularly as they would like, and they do not always obtain the medicines they need [33]. Given that corruption is so widespread in Bosnia, it is expected to be one of the factors that negatively affects access to health care. Despite the fact that the effect of corruption on general access to health care has been explored widely [4,5,6,7], there is little research on how returnees’ access to health care is affected by corruption in the healthcare sector. This issue is of great importance given the increasing number of Bosnian returnees. This study therefore examines how corruption in the healthcare sector affects access to health care for return migrants, based on an analysis of the experiences of Bosnian refugees who have resettled in a country with a high level of corruption after many years of residence in a country with a low level of corruption.

The Bosnian healthcare system is highly decentralized and relies mainly on public providers. Only around 10% of all healthcare employees work in private practices, but this number is increasing. The general practitioners act as gatekeepers, so contact with specialized care cannot be made directly by the patients. All health care is, at least officially, free of charge for individuals older than 65 years. Other patients must pay whenever they seek health care, the fee depending on the medical treatment provided. Thus, out of total expenditure on health, 50% is paid privately. All citizens have the option of purchasing voluntary health insurance. The monthly cost of voluntary health insurance in Bosnia is on average about 5% of the gross salary. The satisfaction with the healthcare system in Bosnia is generally low [34,35,36].

## 2. Materials and Methods

In an earlier article, we explored the driving factors for return migration based on the same empirical study as the present article, and the methodology is therefore described in detail elsewhere [33]. Here we focus on those aspects of the methodology that are particularly relevant to this article. In order to uncover potential issues concerning barriers to access to health care, we interviewed elderly, chronically ill Bosnians who frequently need healthcare services. We conducted 18 semi-structured interviews with 33 individuals who had returned to Bosnia after approximately twenty years of residence in Denmark. The inclusion criteria, besides returnee status, were age (over 55 years) and diagnosis of a chronic illness. The interviewees were recruited with the assistance of a repatriation consultant at the Danish Refugee Council and participants in the study who had already been interviewed, using the snowball method. The consultant had information on individuals’ illness status. Besides the 33 participants, two Bosnian healthcare professionals were interviewed for background information. The interviews took place in the homes of the interviewees in various locations in Bosnia. They were conducted by the first author with the assistance of a Danish-Bosnian interpreter during the winter and spring of 2014. 

Most of the interviews were with married couples, who were interviewed together. This was of great value, as the couples had spent most of their lives together, including their history of flight, refuge in Denmark and return to Bosnia, and they were used to accompanying each other when telling stories from their lives. In the interviews we focused on the participants’ narratives and used an explorative approach in order to uncover factors of key importance in access to health care. Corruption emerged as an important issue in these interviews. Having received a thorough introduction to the study, including the right to withdraw from it at any time, all participants gave verbal consent to participate before the interviews were conducted. Informed consent was given verbally rather than in writing, as this was believed to establish a less formal situation and thereby facilitate a more open dialogue. According to the principles of the American Anthropological Association consent “does not necessarily imply or require a particular written or signed form. It is the quality of the consent, not its format, which is relevant” [37]. After the interview, all participants received a small present as a symbol of gratitude. All informants were ensured anonymity.

All the interviews lasted between 45 min and three hours. Even though some of the interviews were longer than others, a point of saturation with regard to the topic of investigation was generally reached before the interview was completed. Recruitment of interviewees was discontinued when no new themes occurred in the interviews, and the number of interviewees was therefore arrived at during the fieldwork. All the interviews were recorded, transcribed verbatim and translated into Danish from Bosnian. The analysis was inspired by systematic text condensation as described by Malterud, a method that permits an explorative approach to the data [38]. Reoccurring topics were categorised, and relevant quotes were selected and translated from Danish into English by the first-author.

### Characteristics of Participants

The characteristics of the participants are presented in Table 1. All participants had arrived in Denmark as refugees during the 1990s. One couple and one man had returned ten and eleven years respectively prior to the interview, whereas the rest had returned less than four years beforehand as part of the Danish repatriation program, which does not allow re-entry into Denmark after one year’s absence. Except for one spouse who was not ill, all participants were suffering from one or more chronic diseases. None of the chronic conditions were life-threatening at the time of the interviews. Very few had been employed while residing in Denmark, and at the time of the interview they were all unemployed or retired.

The following participants underwent dyadic interviews: 1 and 2, 3 and 4, 6 and 7, 8 and 9, 10 and 11, 12 and 13, 14 and 15, 16 and 17, 18 and 19, 20 and 21, 22 and 23, 24 and 25, 27 and 28, 29 and 30.

## 3. Results

In the following, findings from the interviews with the Bosnian returnees relating to how corruption in the Bosnian healthcare sector affects access to healthcare services will be presented. In general, corruption in Bosnia was frequently experienced by participants, and it was also described as being strongly present in the healthcare system. Corruption occurred in the form of bribery, gift-giving and special favors to friends and relatives, and the returnees described how, in different ways, these practices constituted a barrier to their access to health care.

### 3.1. Experiences with Corruption

Virtually all participants stated that they had had personal experience of corruption in Bosnia. They noted that corruption existed at all levels of public office and that the healthcare sector was no exception. Many of the participants also described having encountered requirements for bribes in various situations, and the politicians were often blamed for this situation. One participant described it this way:

“Politics is rotten to the core. Perhaps you have seen the demonstrations? That’s how far we are. It (demonstrating) should have been done long time ago. Everything here is corrupt. … If you don’t have money, you can’t go anywhere, you can just die. That’s how it is when politics is rotten to the core. And those guys, Bakir (the president) and the others, they steal the most. But it’s all well-known—that I have to pay the doctor, the police officer. Imagine, you can buy a police officer by giving him 10–20 Bosnian Marks (equivalent to 6–11 USD), then you can drive without wheels. That’s the kind of state this is.”

(Participant number 7)

In talking about “the demonstrations”, this participant was referring to the anti-government clashes that took place in February 2014, when people protested against their allegedly corrupt government outside the city halls in numerous cities around the country. Several public buildings, including the presidential building, were partly burned down, and the demonstrators demanded that corrupt politicians step down. The demonstrations took place after several state-owned companies had been sold to private individuals who had personal connections with those who facilitated the sales, and the privatizations meant that many public employees were fired.

This participant experienced obstacles and corruption in many aspects of his life. He and his wife had returned to Bosnia to live with their son and his family in a small village. They received a monthly payment from Denmark but, apart from that, their son was the sole breadwinner in a family of three generations. This son, however, only received his salary every third or fourth month, leaving the whole family in an economically precarious situation. According to the participant, his son’s employer did not like him, and as the employer had the power to express his dislike by not paying the son regularly, he did so. The participant further stated that he was frequently asked to bribe public officials in order to obtain services from them or to avoid being fined by policemen. He described having encountered many situations in which he had to submit to demands for bribes from people who were in higher-ranking positions in the social order. He expressed great frustration at this, explaining that it had reached the point where he had given up fighting against it; he had accepted that there was no other way than agreeing to giving bribes in order to get what one needed. This did not mean that he succeeded in this; he and his wife both suffered from chronic pain and therefore needed frequent pain relief treatments. They often had to manage without them, however, as they could not afford to bribe the doctors sufficiently.

Several other participants described having had similar experiences with bribery in the healthcare system; typically they were asked to pay extra for services at the doctor’s or they were forced to pay in order to be moved up a long queue of patients. Very rarely did they receive any receipts for such payments, suggesting that this money was meant for the doctor himself, not for the clinic. Many participants shared this view. When we asked one participant—who suffered from cardiovascular disease and hence needed frequent check-ups at the doctor’s—whether he had experienced any corruption in Bosnia, he replied “Always. The first thing the doctor looks for is what he can put into his bag.” (Participant number 9)

Even though there was widespread corruption in the healthcare system in the form of doctors who, in their role as public officials, asked for bribes and thereby misused or abused their position of power for private gain, not all participants condemned corruption as strongly as the participants cited above. The following section describes the practices of participants whose approach to corruption was less negative.

### 3.2. Gift-Giving

Those who did not condemn corruption strongly seemed to have adapted differently to the system by viewing it as a part of a broader system of inter-personal relations of exchange. It was, for example, common for them to engage in what they called gift-giving practices that involved not only money, but also food, flowers, cosmetics and other items. Some Bosnian returnees described such gift-giving as voluntary and emphasized that it was a natural thing to do in order to show appreciation for what the doctor had done. As one participant, who suffered from diabetes and who had large expenses in connection with his treatment, put it:

“My wife and I, we always give tips. … As you know, we people from the Balkans, we do not permit the doctor to do anything without us buying him a gift.”

(Participant number 28)

Giving gifts to the doctor seemed to establish a personal relationship between doctor and patient, which initiated a mutual feeling of responsibility benefiting the latter. However, gift-giving did not always take place out of pure appreciation; it was also described as a crucial way of securing quick and friendly treatment in the future because it would make the doctor think of the patient as deserving of good service. Some participants even claimed that they feared not being treated well in the future if they did not give gifts after a treatment. The same participant, who emphasized the importance of tipping, described his gift-giving in connection with surgery of gallstones in Bosnia as follows:

“I gave the doctor 100 Euro (equivalent to 111 USD). In this way I knew that the doctor would help me if I needed it. The doctor thinks better of you if you pay him; he takes better care of you if you have given him a present. … If there’s a need for a check-up after surgery, for example, it normally takes between six months and a year before you get it. But if you pay, it is much quicker; then it takes one month, two months or maximum three months before the doctor will see you again.”

(Participant number 28)

Hence, giving a gift to the doctor after successful surgery in order to secure timely and good treatment in the future is often regarded as a natural aspect of expedient patient-doctor relations in Bosnia, whereas this would normally be regarded as inappropriate in Denmark, though it does happen occasionally. Giving money is, however, not accepted in Denmark. Indeed, one participant explained that she was warned against giving Danish doctors money, even though she would have liked to do so in order to thank them for successful surgery:

“In Denmark, the system is good. He (her husband) had surgery on his back, and we asked one of our doctors: “What are we supposed to do?” He (the doctor) said: “Under no circumstances can you give money, you would be told off.” So we didn’t dare to do so.”

(Participant number 27)

Gift-giving was a strategy used by many of the returnees to navigate their way through the corrupt healthcare system, and giving gifts, including money to the doctor seemed to make for easier access to health care. Indeed, those who gave gifts to doctors did not express as much frustration about the corruption in the system as those who did not.

### 3.3. Size of Bribes

The distinction between gifts and bribes is by no means clear-cut [23]. We have chosen to refer to bribes when the situation involves healthcare personnel’s more or less direct requests for extra payment, whereas we refer to gifts when the patients give objects or money without directly being requested to do so.

According to the participants, the size of the bribe the doctor requested depended on how much he or she thought the patient was able to give. Similarly, the amount of money the patients offered to the doctors varied according to how much they could spare. As one couple put it, when asked how much money patients “normally give as a present”:

“That depends on how much you are able to give.”

(Participant number 28)

“100 Euros, 50 Euros (111 USD, 56 USD, respectively) It depends on how much you have. The person who has more gives more.”

(Participant number 27)

The demand for extra payment turned out to be an important barrier to accessing health care for the many returnees who had limited economic means, preventing them from obtaining the health care they needed. When we asked one participant, who lived alone and who had never had a regular income, whether she gave the doctor extra money to ensure that she received the required treatment for her diabetes, she replied:

“From where, from where would I get the money? I have to live, so I can’t pay extra.”

(Participant number 29)

As a consequence, she only received some of the diabetes treatment she needed. Aggravating the problem was also the fact that returned refugees were generally regarded as being wealthier than those who had not left Bosnia during the war. It was a common view in Bosnia that returnees received large amounts of money from abroad, and many doctors therefore demanded larger sums from them than from others. As noted by one of the returnees, who suffered from chronic pain and lived primarily on the repatriation support he received from Denmark, since his family’s farming did not provide them with any income:

“It’s a big issue that when they find out that we have been abroad … then they increase the size of the payment. They then think that we have money, but we only receive 700 Bosnian Marks (399 USD) per month.”

(Participant number 19)

The doctors’ assessments of each patient’s wealth therefore often did not match the patient’s economic resources.

It should be noted that due to the repatriation legislation, it was not allowed for the Bosnians to seek health care in Denmark after they had returned to Bosnia. The participants thus had no option of using the Danish healthcare system occasionally, even though they would have wanted to supplement the health care they obtained in Bosnia with other services.

### 3.4. Favors from Friends and Relatives

Apart from gift-giving, the Bosnian participants also sought to improve their access to healthcare services by drawing on their networks of friends and relatives. Favors from friends and relatives, who were either employed in the healthcare system or who knew someone who was, offered a way to avoid the demands for bribes and doctors’ expectations of gifts. Thus, having good connections in the healthcare system and using them was known to provide easy and often less expensive access to services. Several participants emphasized that knowing the doctor could lead to exemption from paying bribes and to being able to jump the queue, and they described how this method was very commonly used in Bosnia. However, only a minority of the returnees interviewed in this study knew anyone in the healthcare system they could ask for favors, and they therefore only rarely benefitted from special treatment from friends or relatives. On the contrary, they were at a disadvantage compared to individuals who had not been away from the country. During their long period of absence from the country, many returnees lost the connections they had had before they left Bosnia.

An example of a woman who had no connections who could help her in obtaining access to healthcare services was participant number 33, a formerly very well-connected woman who suffered from arthritis and cardiovascular disease. She had worked for 35 years at a Bosnian hospital before she lost her job and fled the country. Through her job she had known and frequently obtained assistance from many healthcare workers, but after her return she found that everybody she used to know had either left or died. She therefore had no one to assist her in obtaining care upon her return, and she explained that this meant she would now only seek health care as a last resort, whereas before she fled the country she used to obtain health care whenever she needed it.

The returnees’ lack of connections thus compounded the difficulties they experienced in obtaining access to health care. If they could not pay the expected gift of money and had no well-connected family and friends, they would remain at the back of the queue.

## 4. Discussion

Many of the Bosnian returnees interviewed for this study described encountering a high level of corruption in the healthcare sector in Bosnia. However, despite the barriers that corruption caused, some interviewees seemed to have given up opposing it. This can be explained by the fact that corruption is systemic in Bosnia and that it to a certain degree therefore has become a “standard operating procedure” in the sense that “people interpret life in terms of corruption” [39] (p. 103). In other words, people expect to be met by corruption in their encounters with public officials and perhaps with others in general. They therefore do not always actively resist corruption, but rather try to cope with it in a pragmatic manner. However, unlike those who never leave their country of origin and therefore continue to live with a corrupt system, returnees who have become used to living in a country with little if any corruption, no longer (to the same extent) consider corruption to be a natural part of the system. Unlike those who stayed behind, they may find corruption less natural to live with and cope with. An example of this can be found in Ilkjaer’s study of highly skilled Indians who returned to India after living and working for a number of years in Western societies with little corruption. When, upon their return, they encountered corruption among public officials, they needed to realize, and adapt themselves to, the fact that bribery was still very present in India. This was difficult for many, as they had come to view bribery as morally wrong, leading them to feel alienated in what they regarded as their home country [40]. Similarly, Paasche’s study of Iraqi returnees found that they had adapted to a less corrupt society abroad and that the corruption in the home country therefore made them feel a sense of alienation from their home country. Paasche describes how the returnees considered corruption a major challenge to their own reintegration both psychosocially, by creating a feeling of insecurity and hindering the development of a sense of belonging in their home country, and economically, by obstructing entrepreneurship and producing relative deprivation [41]. Corruption has thus been shown to create complications for return migrants other than the Bosnians in this study.

Our interviewees similarly described how corruption led to problems with reintegration in terms of distrust towards authorities, despair at the immoral practices in the government and a general feeling of alienation. They were keenly aware that nepotism played a key role when jobs were filled, that roads were only well-maintained in areas with ethnic majority populations with good connections to the government, and that there were huge gaps between the living conditions of public officials and ordinary citizens respectively. Many were frustrated and worried by this situation and felt stressed and insecure about the future. Corruption therefore impacted negatively on their well-being. In combination with the restrictions in access to healthcare services it created, corruption therefore had a strongly adverse effect on their health status. The interviewees’ motives for returning, despite the levels of corruption they would encounter, is explained by their desire to return to where their families were and to where they felt a sense of belonging [33].

According to the United Nations Office on Drugs and Crime, the size of an average bribe paid in Bosnia is 125 USD [26]. The present study shows that the size of bribes varies a lot, depending on what the doctor expects the patient to be able to pay, and also on how much the patient thinks he or she can spare. Furthermore, those who did not leave the country during the war might have felt betrayed by the returnees for having left the country during a period of hardship. This might have given them an incentive to punish the returnees or at least to not shelter them from exploitation, and the doctors did this by requesting larger bribes. The general negative and often discriminatory response to returnees has been described by Stefansson as being widespread in post-war Bosnia [42]. Hence the size of the bribe is ambiguous and to some degree negotiable. As described, the Bosnian returnees are often regarded as being wealthier than non-returnees and they are therefore asked for larger bribes. As mentioned, the Bosnians do receive a monthly payment from Denmark after their repatriation; however, this payment is only marginally larger than the average income in Bosnia, and does not necessarily allow them to pay larger bribes. A similar situation has been described by Paasche, who studied the return of Iraqi refugees. Paasche reports that Iraqi refugees who had returned from Norway and the United Kingdom were more frequently asked for bribes than was the case for those who had never fled from Iraq, and they were also asked for larger bribes than individuals who had not been refugees abroad [41].

The widespread corruption in Bosnia stands in stark contrast to the low level of corruption in Denmark. The occurrence of corruption generally varies considerably from country to country across the world [21]. While different reasons for this variation have been presented by various scholars, no consensus focusing on one major cause of corruption has been reached [8]. Countries with high levels of corruption, however, share certain characteristics. For example, countries in Central and Eastern Europe, which undergo the transition from communism to capitalism, are characterized by being low in interpersonal and generalized trust, as is generally the case with communistic systems. One explanation for the low level of trust in transitional countries is an increase in economic inequality. Trust seems to be a stable phenomenon in the sense that the presence, or lack, of trust does not change much over time. As trust and corruption levels are inversely associated, the low level of trust linked to transition and a former communistic system can be important explanations for why there is such a high level of corruption in Bosnia [43]. Corruption has, more specifically, been shown to be correlated with lack of trust in public officials and institutions. In societies with high levels of corruption, people have low levels of trust in public officials and institutions, but high levels of trust in their peers, such as friends and family. According to a study by Nannestad et al., there is a low level of trust in institutions in Bosnia, whereas the level of trust in Danish institutions is high [44]. It seems that the low level of trust in Bosnia is compensated by high levels of trust in personal social relations with, for example, one’s doctor, leading to traditions of gift-giving. The high level of trust in institutions in Denmark, on the other hand, makes personal social relations with people in public positions unnecessary and therefore gift-giving redundant. The fact that our participants considered giving gifts to healthcare personnel in Denmark, even though this is generally not the custom there, shows that it takes time to adjust to new norms in this regard.

Contrary to the lack of consensus on what causes a society to be corrupt, there is general agreement that the variation in occurrence of corruption is not caused by dissimilarities in the acceptance of corruption among different populations. In fact, people in different cultures seem to share a very similar notion of what should count as corruption, and they condemn the same practices as corrupt and as morally wrong [45]. This is confirmed by the fact that doctors’ demands for bribes from patients seem to be regarded as corruption in both Bosnia and in Denmark. However, when it comes to giving gifts, there is a difference in perceptions between the two countries. In Denmark, giving gifts to a doctor is to a large extent regarded as an inappropriate act, whereas some Bosnian returnees described gift-giving as voluntary and as a natural thing to do in order to show appreciation for what the doctor has done. As there was this difference in how gift-giving was perceived, this study cannot confirm the theory of a similarity in the acceptance and condemnation of specific acts as corrupt.

This study has explored the way corruption in the healthcare sector affects return migrants’ access to health care, a topic that is rarely touched upon in the literature. By focusing on return migrants who have been moving between countries with very different levels of corruption, the nuances in the consequences appear clearer. While the number of interviews conducted was relatively small, most of the interviews were with couples and thus examined the experiences of two individuals. In this way this approach made it possible to investigate a greater range of experiences on the basis of more nuanced accounts than a larger number of interviews with individuals would have enabled. When using the snowball method to recruit participants, there is a possibility that selection bias occurs because the participants may come from the same socio-economic milieu and thereby represent similar characteristics and experiences. If recruitment had instead followed a maximum variation technique, the participants probably would have displayed greater socio-economic differences [46]. However, since corruption is so widespread in Bosnia, we expect all socio-economic segments of the population to be exposed to the corrupt practices. Furthermore, the better-off can be expected to be met with requests for larger bribes than those who are economically constrained (in accordance with the larger requests that return migrants encountered compared to non-returnees). We therefore do not expect the findings of this study to differ substantially from the findings that would have resulted from a study employing another recruitment method. Limitations connected to the use of an interpreter in interview situations are widely known [47]. We acknowledge that nuances generally may have been lost due to the usage of an interpreter; however, we also acknowledge the positive effect in terms of the cultural sensitivity provided by the presence of a Bosnian interpreter acquainted with local conditions.

Much emphasis in research and policy development has been placed on exploring ways to prevent corrupt practices and to put an end to corruption in health care and in general [4,9,17,39]. This work is very important, and support for this, as well as for local networks and civil societies fighting for corruption-free systems, should continue. The fight could be strengthened by involving diaspora associations, as diaspora migrants are recognized as agents of change in the sense that they transfer knowledge, skills and ideas to individuals in their country of origin [48]. As this study has shown, migrants living abroad have often taken a more critical stand towards corrupt practices, and they are therefore often very eager to fight corruption. However, as combatting corruption has proved very difficult, if not impossible, we further suggest the establishment of networks and organizations that can help the returnees obtain access to healthcare services legally upon their return. These organizations could initially focus on helping people in serious need of health care, such as the elderly and chronically ill, as a means of limiting the inequality in access to health care that corruption entails. Though this only constitutes symptom management and not a cure, it could improve the health conditions for this vulnerable group.

## 5. Conclusions

This study has found that repatriated Bosnians experienced corruption as being widespread in the Bosnian healthcare sector. To some degree they accepted the high level of corruption and the accompanying requests for bribes and expectations of gifts from doctors and to some extent learned to live with it. However, in comparison to those who have never left the country, the returnees faced greater problems, as the doctors expected larger bribes from them, and they also lacked connections among friends and relatives who could help them avoid corrupt practices. The corruption in the healthcare sector thus created a barrier that limited the returnees’ access to the healthcare services they required. This was a serious problem for chronically ill individuals who were generally in need of frequent health care, which ultimately affected their health negatively.

## Figures and Tables

**Table 1:** Characteristics of informants.

Participant Number	Age *	Sex	Years Since Return	Illness	Educational Level **	Employment in Bosnia before Flight
1	3	Female	1	CVD	2	Cashier
2	3	Male	1	CVD	2	Electrician
3	1	Female	2	Diabetes, asthma	1	Housewife
4	2	Male	2	Diabetes	2	Workman
5	3	Male	2	Psoriasis	2	Electrician
6	1	Female	3	Chronic pain	1	Housewife
7	1	Male	3	Chronic pain	2	Barrel maker
8	2	Female	4	Diabetes	Unknown	Housewife
9	2	Male	4	CVD	Unknown	Odd-job man
10	2	Female	10	CVD	2	Finance manager
11	1	Male	10	Diabetes	2	Purchasing agent
12	2	Female	1	CVD	1	Housewife
13	2	Male	1	Chronic pain	1	Mechanic
14	1	Female	2	Chronic pain	2	Seamstress
15	2	Male	2	PTSD	2	Office worker
16	2	Female	4	Chronic pain	1	Housewife
17	2	Male	4	Chronic pain	1	Labourer
18	2	Female	1	Diabetes	1	Chicken farmer
19	2	Male	1	Chronic pain	1	Bricklayer
20	3	Female	3	Chronic diarrhoea	1	Housewife
21	3	Male	3	CVD, Parkinson’s disease	2	Fireman
22	2	Female	3	Diabetes	1	Cashier
23	2	Male	3	PTSD	1	Mechanic
24	1	Female	4	Diabetes	1	Referent
25	1	Male	4	Chronic pain	1	Shop manager
26	3	Female	3	Diabetes	1	Housewife
27	1	Female	3	None	1	Housewife
28	2	Male	3	Diabetes	1	Manager
29	3	Female	3	Diabetes and arthritis	1	Housewife
30	3	Male	11	CVD	1	Butcher
31	2	Male	1	Diabetes	Unknown	Carpenter
32	2	Female	3	Diabetes	Unknown	Housewife
33	3	Female	3	CVD and arthritis	2	Laboratory technician

CVD: Cardiovascular disease. PTSD: Post-traumatic-stress disorder. * Age in years: 1 = 55–65, 2 = 66–75, 3 = 76–85. ** Educational level: 1 = primary and lower secondary school, 2 = youth education, 3 = higher education.

## References

[1] Ricketts T.C., Goldsmith L.J. (2005). Access in health services research: The battle of the frameworks. Nurs. Outlook.

[2] Dixon-woods M., Cavers D., Agarwal S., Annandale E., Arthur A., Harvey J., Hsu R., Katbamna S., Olsen R., Smith L. (2006). Conducting a critical interpretive synthesis of the literature on access to healthcare by vulnerable groups. BMC Med. Res. Methodol..

[3] Andersen R.M. (1995). Revisiting the behavioral model and access to medical care: Does it matter?. J. Health Soc. Behav..

[4] Ensor T. (2004). Informal payments for health care in transition economies. Soc. Sci. Med..

[5] Falkingham J. (2004). Poverty, out-of-pocket payments and access to health care: Evidence from Tajikistan. Soc. Sci. Med..

[6] Ensor T., Savelyeva L. (1998). Informal payments for health care in the former Soviet Union: Some evidence from Kazakstan. Health Policy Plann..

[7] Bouchard M., Kohler J.C., Orbinski J., Howard A. (2012). Corruption in the health care sector: A barrier to access of orthopaedic care and medical devices in Uganda. BMC Int. Health Hum. Rights.

[8] Rothstein B., Rothstein B. (2011). Corruption: The killing fields. The Quality of Government.

[9] Transparency International (2006). Global Corruption Report 2006.

[10] Witvliet M.I., Kunst A.E., Arah O.A., Stronks K. (2013). Sick regimes and sick people: A multilevel investigation of the population health consequences of perceived national corruption. Trop. Med. Int. Health.

[11] Holmberg S., Rothstein B. (2011). Health economics, policy and law. Health Econ. Policy Law.

[12] Kawachi I., Kennedy B.P., Lochner K., Prothrow-stith D. (1997). Social capital, income inequality and mortality. Am. J. Public Health.

[13] Marmot M., Wilkinson R. (2006). Social Determinants of Health.

[14] Wilkinson R., Pickett K. (2010). The Spirit Level: Why Equality Is Better for Everyone.

[15] Rothstein B. (2013). Corruption and social trust: Why the fish rots from the head down. Soc. Res..

[16] Rothstein B., Rothstein B. (2011). What is quality of government?. The Quality of Government.

[17] United Nations Development Programme (2011). Fighting Corruption in the Health Sector Methods, Tools and Good Practices.

[18] Mostert S., Njuguna F., Olbara G., Sindano S., Sitaresmi M.N., Supriyadi E., Kaspers G. (2015). Corruption in health-care systems and its effect on cancer care in Africa. Lancet Oncol..

[19] Berger D. (2014). Corruption ruins the doctor-patient relationship in India. BMJ.

[20] Rahmani Z., Brekke M. (2013). Antenatal and obstetric care in Afghanistan—A qualitative study among health care receivers and health care providers. BMC Health Serv. Res..

[21] Corruption Perceptions Index 2015, Transparency International. http://www.transparency.org/cpi2015.

[22] Balabanova D., Mckee M. (2002). Understanding informal payments for health care: The example of Bulgaria. Health Policy.

[23] Moldovan A., De Walle V. (2013). Gifts or bribes? Attitudes on informal payments in Romanian Health Care. Public Integr..

[24] Delcheva E., Balabanova D., Mckee M. (1997). Under-the-counter payments for health care: Evidence from Bulgaria. Health Policy.

[25] United Nations Development Programme (2002). Human Development Report—Bosnia and Herzegovina.

[26] United Nations Office on Drugs and Crime (2011). Corruption in Bosnia and Herzegovina: Bribery as Experienced by the Population.

[27] Transparency International (2014). Bosnia and Herzegovina: Overview of Political Corruption.

[28] United Nations High Comissionary for Refugees, Bosnia and Herzegovina Regional Office. http://reporting.unhcr.org/node/12002#_ga=1.19672248.146717335.1471518824.

[29] Grünenberg K. (2006). Is Home Where the Heart Is, or Where I Hang My Hat?. Ph.D. Thesis.

[30] Statistikbanken, Statistics Denmark. http://www.statistikbanken.dk/.

[31] The Repatriation Act, Law No. 1099. https://www.retsinformation.dk/forms/r0710.aspx?id=164351.

[32] World Databank: World Development Indicators 2014. http://databank.worldbank.org/data//reports.aspx?source=2&country=BIH&series=&period.

[33] Handlos L.N., Olwig K.F., Bygbjerg I.C., Kristiansen M., Norredam M.L. (2015). Return migration among elderly, chronically ill Bosnian refugees: Does health matter?. Int. J. Environ. Res. Public Health.

[34] Slipicevic O., Malicbegovic A. (2012). Public and private sector in the health care system of the Federation Bosnia and Herzegovina: Policy and strategy. Mater. Soc. Med..

[35] International Organization for Migration (2014). Country Fact Sheet—Bosnia and Herzegovina.

[36] Krupic F., Krupic R., Jasarevic M., Sadic S., Fatahi N. (2015). Being immigrant in their own country: Experiences of Bosnians immigrants in contact with health care system in Bosnia and Herzegovina. Mater. Soc. Med..

[37] American Anthropological Association’s Principles of Professional Responsibility. http://ethics.aaanet.org/category/statement/.

[38] Malterud K. (2012). Systematic text condensation: A strategy for qualitative analysis. Scand. J. Public Health.

[39] Rothstein B., Rothstein B. (2011). Curbing corruption: The indirect “Big Bang” approach. The Quality of Government.

[40] Ilkjaer H. (2015). Bangalore Beginnings—An Ethnography of Return Migration among Highly Skilled Indians. Ph.D. Thesis.

[41] Paasche E. (2016). The role of corruption in reintegration: Experiences of Iraqi Kurds upon return from Europe. J. Ethn. Migr. Stud..

[42] Stefansson A.H., Markowitz F., Stefansson A.H. (2004). Sarajevo suffering: Homecoming and the hierarchy of homeland hardship. Homecomings: Unsettling Paths of Return.

[43] Rothstein B., Rothstein B. (2011). The low trust-corruption-inequality trap. The Quality of Government.

[44] Nannestad P., Svendsen G.T., Dinesen P.T., Sønderskov K.M. (2014). Do institutions or culture determine the level of social trust? The natural experiment of migration from non-western to western countries. J. Ethn. Migr. Stud..

[45] Rothstein B., Rothstein B. (2011). Quality of government: What you get. The Quality of Government.

[46] Christensen U., Schmidt L., Dyhr L., Vallgårda S., Koch L. (2008). The qualitative research interview. Research Methods in Public Health.

[47] Wallin A.M., Ahlström G. (2006). Cross-cultural interview studies using interpreters: Systematic literature review. J. Adv. Nurs..

[48] Danstrøm M.S., Kleist N., Sørensen N.N. (2015). Somali and Afghan Diaspora Associations in Development and Relief Cooperation.

